# A Nano Risk Governance Portal supporting risk governance of nanomaterials and nano-enabled products

**DOI:** 10.1016/j.csbj.2025.06.024

**Published:** 2025-06-13

**Authors:** Panagiotis Isigonis, Evert A. Bouman, Dimitra-Danai Varsou, Keld Alstrup Jensen, Wouter Fransman, Damjana Drobne, Blanca Pozuelo Rollon, Arantxa Ballesteros, Isabel Rodríguez-Llopis, Arto Säämänen, Antreas Afantitis

**Affiliations:** a Ca’ Foscari University of Venice, Department of Environmental Sciences, Informatics and Statistics (DAIS), Via Torino 155, Venice 30172, Italy; b Luxembourg Institute of Science and Technology, Environmental Sustainability Assessment and Circularity, 5, Avenue des Hauts-Fourneaux, Esch-sur-Alzette 4362, Luxembourg; c NILU, Environmental Impacts & Sustainability, Kjøpmannsgata 8, Trondheim 7013, Norway; d NovaMechanics MIKE, Department of Chemoinformatics, Piraeus 18545, Greece; e National Research Centre for the Working Environment (NRCWE), Lersø Parkalle 105, Copenhagen 2100, Denmark; f TNO, Healthy Living & Work, Princetonlaan 6, Utrecht 3584 CB, the Netherlands; g University of Ljubljana, Department of Biology, Jamnikarjeva 101, Ljubljana 1000, Slovenia; h Technological Institute of Packaging, Transport and Logistics (ITENE), Parque Tecnológico, C/ Albert Einstein, 1, Paterna, Valencia 46980, Spain; i GAIKER Technology Centre, Basque Research and Technology Alliance (BRTA), Parque Tecnologico de Bizkaia, ed 202, Zamudio, Bizkaia 48170, Spain; j Finnish Institute of Occupational Health (FIOH), P.O. Box 40, FI-, Tampere 00032, Finland; k VTT Technical Research Centre of Finland, Clean Air Technologies, Visiokatu 4, P.O. Box 1300, Tampere 33101, Finland; l NovaMechanics Ltd., Department of Chemoinformatics, Nicosia 1070, Cyprus; m Entelos Institute, Division of Data Driven Innovation, Larnaca 6059, Cyprus

**Keywords:** Risk Governance, Risk Governance Portal, Nanomaterials, Advanced Materials

## Abstract

Risk governance (RG) of nanomaterials (NMs) has been at the focus of the Horizon 2020 Programme of the European Union, through the funding of three research projects (Gov4Nano, NANORIGO, RISKGONE). The extensive collaboration of the three projects, in various scientific topics, aimed to enhance RG of NMs and provide a solid scientific basis for effective collaboration of the various types of stakeholders involved. In this paper the development of a digital Nano Risk Governance Portal (NRGP) and associated information technology (IT) infrastructure supporting the risk governance of (engineered) nanomaterials and nano-enabled products, is presented, alongside considerations for future work and enhancement within the domain of Advanced Materials (AdMa). This paper describes several elements of this digital portal, which serves as a single-entry point for all stakeholders in need of, or interested in, nano-risk governance aspects. In its simplest form, the NRGP allows users to be efficiently guided towards tailored information about nanomaterials, risk governance concepts, guidance documents, harmonized methods for risk assessment, publicly accessible data, information and knowledge, as well as a directory of tools, to assess the exposure and hazard of nanomaterials and perform Safe-and-Sustainable-by-Design (SSbD) assessment in the context of nano-risk governance. This paper presents the technical implementation and the content of the first version of the NRGP alongside the vision for the future and further plans for development, implementation, hosting and maintenance of the NRGP aimed at ensuring its sustainability. This includes a procedure to link to, or include, currently available and future (nano)material-related (cloud) platforms, decision support systems, tools, guidance, and databases in line with good governance objectives.

## Graphical Abstract

**Figure:**
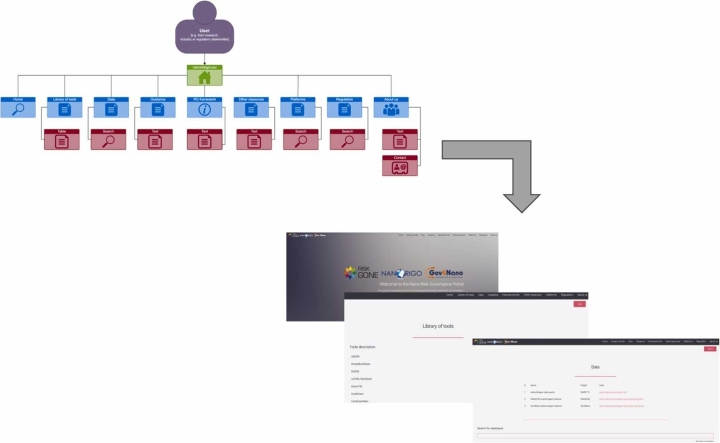

## 1 Introduction

Nanotechnology is a key enabling technology (KET) [1] that can bring large benefits to society – either by improving the existing functionality of currently available materials or adding completely new functionality in nano-enabled materials. As part of the Advanced Materials (AdMa) group, they belong to a large category of materials that are either being developed or have recently been introduced to the market to solve societal challenges [2]. These materials support the strategic and scientific vision of the European Commission and the Green Deal [2]. However, nanomaterials have also been identified to pose potential hazards to human health and the environment, that go beyond the hazards commonly associated with chemicals [3], [4], [5]. Such negative side effects will need to be minimized and balanced against the benefits. In addition to chemical effects, their size and shape may allow them to interact with their surroundings in ways requiring special attention. Therefore, responsible and sustainable use and development of nanomaterials, and nano-enabled products, require a balance between benefits and risks that should include a broad societal inclusion as well as timely research and standardization to support regulation [6], [7], [8], [9].

Recognizing this, a series of research projects, both within and outside Europe, have delivered a significant corpus of nano-related research in the past two decades, covering elements ranging from datasets on physicochemical characterisation, release, exposure, biological responses including biocompatibility, hazard and safety to new internationally accepted and validated testing guidelines, and complete risk assessment, evaluation, and risk management frameworks. However, scientific progress solely is not enough to address the needs and uncertainties of society regarding new technologies. We believe this can only be done by establishing a common approach grounded in a risk governance framework and bringing together stakeholders representing various dimensions in society [10], [11]. A framework [12] for risk governance of nanomaterials is considered an essential element that serves as a framing concept and an actual set of practice, which brings together the totality of actors involved in Risk Governance of nanomaterials [10], [13]. Risk governance frameworks for nanomaterials have been analysed and reviewed in recent scientific studies with the aim to move towards operationalisation and expansion to adjacent research topics [8], [11]. An operational, trans-disciplinary Nanotechnology Risk Governance Framework (NRGF) that integrates exposure, hazard and risk assessment tools with those assessing ethical, legal, social, and environmental aspects and, further supports responsible research and innovation (RRI), has been elaborated throughout the three projects implementation period [14]. A nano-risk governance framework (NRGF) is meant to be a basis for risk-related social/reflective multi-stakeholder discourse [15]. Hence, the NRGF should be operationalised to enable discursive engagement between multiple stakeholders, including non-scientists and interested members of the general public that is based on equal access to nano-risk-related resources.

Risk governance seeks to include all actors, rules, conventions, processes and mechanisms involved in the collection, analysis, communication, and management of, and decisions based on, risk information [16], [17], [18]. It involves multiple stakeholders and considers the wider legal, political, economic, and social contexts in which risks are evaluated and managed. It pertains to the methodologies and processes used to formulate and implement risk-related decisions. Decisions should be based on a clear understanding of risk and a broad and focused interaction between all stakeholders involved, including experts, regulators, industries, and civil societies [19]. It is here where risk governance comes into play, to deal responsibly with uncertain, complex and/or ambiguous risks. However, stakeholders at present have difficulty finding their way in the complicated landscape of information on risks to human health and the environment associated with nanomaterials, and hence would benefit from guidance towards high-quality information, methods and tools to meet their needs. Furthermore, the challenges faced with risk governance of nanomaterials are considered directly relevant for the risk governance of AdMa, which could greatly benefit from lessons learned from risk governance of nanotechnology [7], [10].

Within the EU H2020 programme, the joint efforts from three projects (Gov4Nano,^1^ NANORIGO,^2^ RISKGONE^3^) aimed to support risk governance processes through the development of a Nano Risk Governance Portal (NRGP), allowing users an oversight of available data and tools for managing possible risks of nanomaterials. Earlier examples of such portals or digital infrastructures were developed in a range of previous research projects (see e.g., the web infrastructures developed by the EU projects NanoCommons,^4^ caLIBRAte^5^ and the catalogue of services of the EC4SafeNano Centre [20]), as well as at international organisations (see e.g., the nanotechnology knowledge base from the EU Observatory for Nanomaterials - EUON^6^). However, one single digital infrastructure web portal, serving as a one-stop-shop with all these elements was not available. While the caLIBRAte portal was used as a starting point for one of the three projects (i.e., Gov4Nano) to establish a platform with a regulatory scope, the nano-risk governance science information is wider and in continuous development across numerous organisations and research projects. Better findability and access to the comprehensive volume of nano-risk governance information could improve the use and implementation of information and (software) tools, supporting the implementation of the FAIR principles [21], [22], including in research software [23]. The implementation of the NRGP, as presented in this article, has the ambition to support stakeholders to overcome the aforementioned obstacles by aiding users in finding relevant risk governance information for their nanomaterial decision-making process, while also establishing a path for the development of similar solutions for AdMa.

This paper aims to outline the layout of the NRGP as an IT infrastructure informing on and supporting the risk governance of nanotechnologies and nano-enabled products, both as a source of information and as a gateway to other knowledge resources. This work is the result of a dedicated working group consisting of participants of the three European research projects, working towards the development of the NRGP. In Section 2, we introduce first the methods of designing the NRGP based on a development framework and the exploration of various implementation scenarios. In Section 3, we sketch the chosen implementation scenario and content of the NRGP, and an overview of the current available version of the NRGP. Finally in Section 4 & 5, we address the management of the NRGP and its interplay with other elements developed both within and external to the originating research projects, while addressing issues such as the update of current content and the incorporation of new elements that may become available in the future.

## 2 Methods

This section presents the process behind the conceptualisation and design of the NRGP and digital infrastructure. Starting from a framework for the development of a nano-risk governance portal (hereafter referred to as the development framework) presented in the Section 2.1, we describe the co-creation process in the Section 2.2. Finally, we highlight the development of the chosen solutions in the Section 2.3.

### 2.1 Development framework

The NRGP, as a web portal, is built on the recognition that such an infrastructure can facilitate and support the risk governance of nanotechnologies and guide its users within the existing landscape of datasets, models, tools, guidance, frameworks, and platforms. A main argument for an IT infrastructure to facilitate and support the risk governance of nanomaterials and nano-enabled products is to make the available resources transparent and accessible to all actors (stakeholders). Stakeholders are not always aware of available resources for their development or governance process, nor the value or validity of these resources. For example, regulatory safety assessments use various methodologies and tools, some of which are not applicable to nanomaterials and may lead to incoherent outcomes [24], while state-of-the-art knowledge from academic studies is not sufficiently exploited and reused in governance processes or regulatory risk assessment [11], [25]. Multiple assessments of stakeholders’ needs and priorities through workshops, dedicated meetings and consultations, have been undertaken within the duration of the three research projects [26], identifying expected primary users of the portal as innovators, E&HS (environment & health and safety) personnel and service providers, nano-risk governance specialists, authorities and regulators, and industry managers, which would appreciate a one-stop-shop entity to find or be directed to relevant nano risk governance content [27]. Examples of stakeholders’ needs include but are not limited to accessibility, usability, affordability, extended functionality, reliability and efficient management of available resources, such as tools, safety data and databases [28]. The results of the assessment have been taken into consideration during the development of the NRGP.

The Guiding principles for the development of the functionalities of the portal are described below:

• The portal is a common access point for various user groups and provides a one-stop-shop interface for all resources relevant to nano-risk governance;
• Data, information, and knowledge are resources for any knowledge-based risk governance [29];
• The portal acts as a gateway to specific tools, methods, models, guidance and data (i.e., digital infrastructure);
• Tools, methods, guidance and data are published directly on the portal and the portal can link to other relevant platforms for nano risk governance;

The portal will facilitate access to, dissemination of, and future implementation of new information, data, tools, and guidance to support various user groups in risk governance of nanomaterials and nano-enabled products. It has an ambition to become the most extensive (nano)-material-related repository of resources needed for (nano)material innovation and application activities.

### 2.2 Design process

**Fig. 1:**
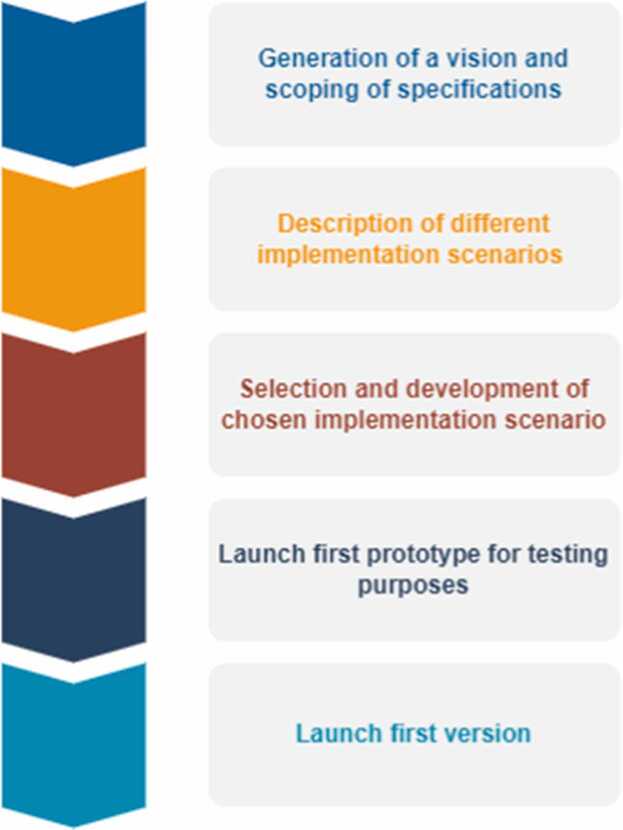
Graphical depiction of the implementation process.

The development framework described above has set the stage for the design process of the portal. Starting from the scoping of a small set of desired specifications and functionality, the implementing working group followed a co-creation process based on a cascade development model. This design process is depicted in Fig. 1. A first prototype of the NRGP, with limited functionality, was developed and launched in June 2022. The working group further developed this prototype into a first public version, available online (www.nanoriskgov.eu) and launched in 24–25th January 2023 during the conference titled “NMBP-13 Future-proof Approaches for Risk Governance – Lessons Learned from Nanomaterials”,^7^, which was held at the OECD premises in Paris and was jointly organised by the three research projects.

The NRGP derives its utility from being a dynamic and easy to update product, i.e., it should be further developed and updated in response to inputs and needs of the nanosafety and AdMa communities in the future.

### 2.3 Development scenarios

In this section, we outline in more detail which options were considered for the implementation of the NRGP as a web portal. The NRGP is intended to be a way of operationalizing the nano risk governance frameworks. Thus, various scenarios were discussed and investigated, mainly distinguishable by the ambition level and difficulty of technical implementation. One key challenge in the development of the portal has been to integrate content from different origins, while at the same time catering to different stakeholders, each with its own desired requirements for content.

The most ambitious and difficult (technically) scenario considered was to implement and offer through the web portal a fully functional suite with interactive data, models, tools, and guidance. Main advantages of such an approach included the possibility for users to stay within an integrated environment for their entire nano governance or research and development process, and even opening up for the possibility of outcome sharing between different tools included in the portal. However, major challenges have been identified in implementing such a solution, as content cannot be easily adapted into the portal due to issues either with functionality and interoperability (e.g., tools are not available as a web service, are coded in various programming languages) or due to the license conditions of preferred material (e.g., commercial and/or protected products cannot be incorporated into the portal). In addition, experienced personnel would be required to build and maintain such a scenario, which is not realistic within the context of research projects with limited duration.

In the current NRGP version, the working group focused on a scenario where the portal is mainly an information resource which is able to guide various user groups with relevant content and linking to specific resources. It is centred around a library of datasets, methods, tools, regulations, guidances and other relevant content. We did not facilitate entries for specific user-profiles and guidance on tools and data selection, but filters that allow users to identify relevant content.

## 3 NRGP development

Having decided on the scenario of the NRGP as an information resource, the technical development from the perspective of digital architecture is presented below. Recognising that the IT infrastructure will have to satisfy many different, and potentially conflicting, needs, the technical development aims to serve these needs with the content of the first version of the portal.

### 3.1 Architecture and content of the nano risk governance portal

**Fig. 2:**
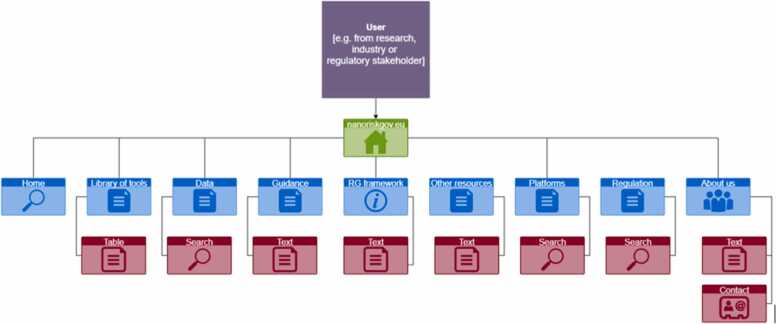
Sitemap sketching the content of the nano risk governance portal.

We sketch the technical architecture of the NRGP and linked instruments for nano-risk governance and incorporating the guiding principles presented in the development framework. Fig. 2 gives an overview of these two elements, in conjunction with other resources that are already available or will be available in the future.

Various user groups are envisioned that interact through the portal, in the context of the risk governance framework, to access tools, data, guidance and methods developed within historical projects. The portal is also prepared to include research outputs developed in future (EU-funded) projects about risk governance and SSbD of nanomaterials or advanced materials. It is focused on providing information, guidance, and a directory with links to relevant tools and datasets for implementation of nano risk governance and SSbD, following different systems.

Within the context of the portal design, we describe the structure for its content in Fig. 2, which indicates the common landing page for different types of users, and subpages on various aspects. At a second level, users access a variety of resources (i.e., platforms, tools, data, guidances, etc.). In addition to these main components, other services and external resources are highlighted. User guidance is provided in the portal to avoid confusion about the content of the various structural levels and to guide the users effectively to the type of resource most adequate to their needs.

**Fig. 3:**
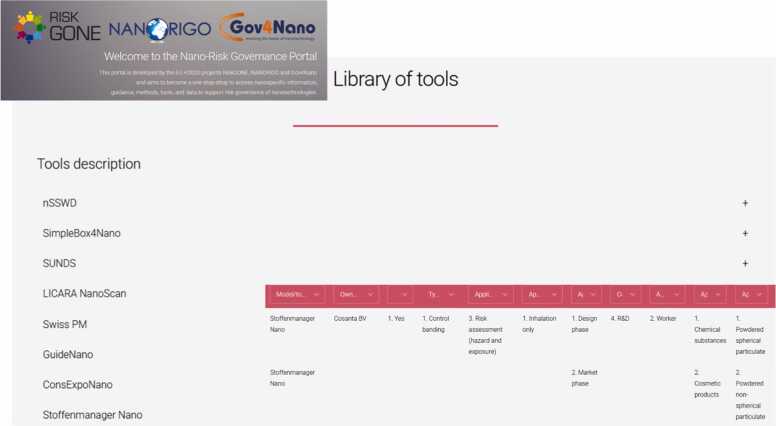
Content impression of the library of tools and the filtering system of the nano risk governance portal, available at: https://www.nanoriskgov.eu.

**Fig. 4:**
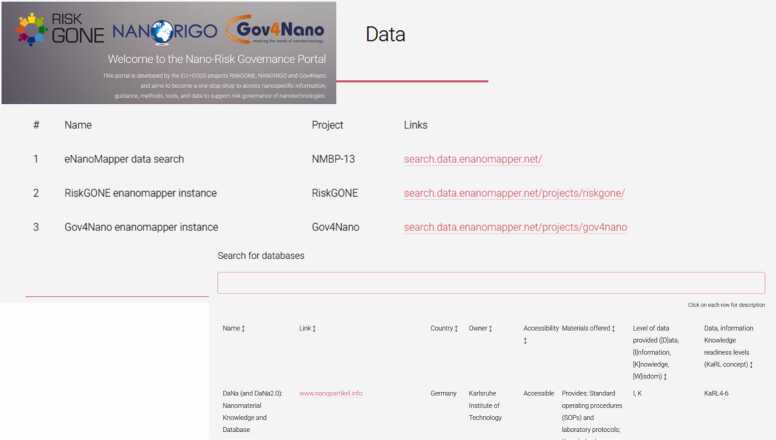
Content impression of the data library and the database search options of the nano risk governance portal, available at: https://www.nanoriskgov.eu.

The library of resources acts as a catalogue of available databases and data repositories, guidance documents and methods, tools and models, information on relevant regulations and various platforms from different projects, all provided with links to the relevant domains. All the sections (e.g., Library of tools - Fig. 3, and Data – Fig. 4) contain curated information that have been made available in close collaboration among teams and working groups of the three research projects responsible for the development of the NRGP, or collected and analysed within each of them. The content of the portal is curated to be relevant to one or more of the stakeholder groups, allowing the easy identification of tailored information. The nano risk governance portal, through its libraries, links to relevant risk governance frameworks and risk governance platforms in a two-fold aim: to provide direct connection to the theoretical background for the interested users, and enable the use of external platforms that provide enhanced functionalities, such as integrated models, databases and tools. In addition, the system is designed to be able to accommodate other historic frameworks, as well as potential future frameworks, platforms, tools and data.

### 3.2 Examples of the use of the NRGP

The portal contains a large amount of information within its inter-related categories. Illustrative examples of use are provided in this section to showcase the functionalities of the portal and how it can be useful for different needs the users may have, while additional detailed information is available at relevant project reports [30].

Example 1 - Aquatic ecotoxicity tests for nanomaterials

A researcher aims to assess the potential environmental impact of a new nanomaterial by evaluating its toxicity in aquatic ecosystems, as part of a new project. Access to the NRGP enables multiple options for the user. Initially, a search of relevant regulation from the dedicated portal page^8^ enables the user to screen related information in a fast and effective way, as a preparatory step, and identify legislation that might be applicable for the study. As the activity involves laboratory testing, the user is aiming to use testing methods aligned with recognised standards, to enhance the reliability and comparability of the study. To facilitate the work and its planning, a visit to the ‘Guidance’ page^9^ allows the user to identify, easily and in very short time, standards applicable and be redirected to the OECD Guidance Document 317 titled «OECD Series on Testing and Assessment No. 317: Guidance Document on Aquatic and Sediment Toxicological Testing of Nanomaterials» . Following a thorough review of the testing procedures, the user may conduct the experiment, ensuring compliance with best practices and regulatory standards. To complement further the study, the user may consult the Data page^10^ to consult databases with existing experimental data or to identify sources to store the new experimental data, ensuring adherence to the FAIR principles. The analysis and interpretation of the test outcomes can be supported with the comparison of findings with existing data. To further assess the risks, a visit to the Library of tools^11^ enables the user to screen through the filters the available tools that can support environmental risk assessment, such as SimpleBox4Nano and the n-SSWD model, and receive a quick overview of description, functionalities, application domains, link to the tools and more.

Example 2 – Navigating the risk governance landscape as a SME

The analysis of stakeholders’ needs showed that SMEs face significant challenges, as they usually lack dedicated resources and in-house expertise for risk assessment and management, while struggling to keep up with rapidly evolving policy and regulation. The portal greatly facilitates the navigation of the risk governance landscape for SMEs by making knowledge, tools, and guidance accessible to organisations of all sizes, in a fast, efficient and cost-effective way. The content is curated and targeted for the various types of users. Navigation is customised and filtering/search options enable the users to assess quickly topics of interest, such as safe handling and use of nanomaterials, regulatory requirements, risk assessment basics, tools availability and more.

A SME representative is interested to evaluate the occupational exposure risks associated with the handling of a nanopowder in its manufacturing process. Similarly to example #1 above, the consultation of regulation, risk governance frameworks, guidance and other sections that might contain relevant information is straightforward. For the specific needs of assessing occupational exposure and risks, accessing the Data^10^ page, provides an overview of databases and repositories of occupational exposure scenarios and tools, such as NECID, the IOM exposure scenario library and the ECETOC Human Exposure Assessment Tools Database, which the SME representative may consult for further information. Furthermore, the use of the ‘applicable population’ filter of the Library of tools page^11^, provides an overview of tools available for assessing occupational exposure, from which the user may select suitable ones, based on the characteristics of the scenario. For example, NanoSafer and SUNDS may be used for performing an evaluation of exposure and risk assessment of nanomaterials, by defining material data (e.g., technical and safety information), processes information (e.g., workplace conditions, protective measures), exposure and hazard data (e.g., measured or modelled) and identify risk control measures.

## 4 Implementation of the NRGP

In the previous sections we have presented the development framework for the NRGP and digital infrastructure, sketched out the technical development and briefly discussed the content of the portal. In the following paragraphs, we address the implementation of the NRGP. Firstly, we discuss the hosting, maintenance and management of the portal considering its intended existence after the end of the originating research projects. Secondly, we discuss the NRGP in relation to, and in interaction with other current, new, and emerging e-infrastructures. Finally, we reflect on the future sustainability of the nano risk governance portal.

### 4.1 Managing the Nano Risk Governance Portal

One challenge with a research-project based effort, such as the NRGP, is that the formal project structures and collaboration between developing partners cease to exist once the projects reach the end of their duration. To resolve this, an agreement has been reached, between the co-authors of the present scientific article, to ensure the continuation of development, allowing for updates of the current content of the portal, and having a mechanism in place to receive new content from the wider stakeholder community.

The portal is currently managed by an international steering committee composed of representatives from the organisations that developed the NRGP and contributed to the collection and curation of all the relevant materials. The steering committee oversees the further development of the portal, through the update of current content, as well as acting as gatekeeper for new content. Further goals of the steering committee are to contribute to the open sharing of data, tools, guidance, and information through the portal, and improve the accessibility to user support and training. As a portal intended to serve the nano-community, the portal is open to contributions from stakeholders and new content may be proposed to the steering committee. Anticipating the establishment of a more formal organisation concerned with the (risk) governance of nanomaterials or advanced materials, the steering committee has the authority to transfer the portal to a legal entity if so desired. Links to existing initiatives (e.g., EU-NSC and INISS) are currently available through the members of the steering committee, while exploration of collaboration with newly established initiatives on Adma or other topics (e.g., IRISS, PARC, IM4EU, AMI2030) will be scoped and evaluated, as those progress with their implementation plans.

The portal developers recognize that the portal is developed by, and based on the results of, projects funded through public resources. In line with the principles of the European Union’s Open Science policy and ambitions [31] and the aim for the portal to be a publicly accessible resource to the nano-community, the portal is therefore licensed under the creative commons license CC-BY-4.0 international. Through this license, it is ensured that the portal can be further developed and that its content may be used widely. Open science aims to ensure the free availability and usability of scholar publications, the data that result from scholar research and the methodologies, including code or algorithms, that were used to generate those data [32].

### 4.2 Interaction with other e-infrastructures

The three research projects at the basis of the development of the NRGP represent 80 organisations, mainly European academic and research institutes, SMEs, industrial partners and NGOs, working on nano-related topics. Nevertheless, the portal is not the only initiative aiming at making information and tools more accessible to the stakeholders of the nano-community, a range of electronic infrastructures is in various stages of development and most likely new e-infrastructures will arise in the (near) future. Notably, there are specific platforms developed within the individual research projects, e.g., the RiskGONE cloud platform as well as the NanoRIGO and caLIBRAte-Gov4Nano platforms that are linked to from the portal already. Furthermore, research projects such as *NanoInformatiX*^12^*, NanoSolveIT*^13^ [33]*, SUNSHINE*^14^*, SBD4Nano*^15^*, SAbyNA*^16^*, HARMLESS*^17^*, SUSNANOFAB*^18^ and others, all develop platforms that could be relevant to be included or linked in the NRGP, as they face similar issues with sustainability and uptake following the end of the project duration [34]. The list of potential e-infrastructures for collaboration includes also past initiatives, such as EC4SafeNano,^19^ which has made available its results for public and free incorporation in the NRGP. These platforms may cover and expand different aspects of nano risk governance, for example the Safe and Sustainable by Design (SSbD) aspects of nano and advanced materials [34]. The nano risk governance portal serves as a one-stop-shop infrastructure to include these other platforms, targeting a wide variety of user groups, and functioning as a gateway to those more specific IT platforms.

Where possible, the steering committee will ensure that links are made between these e-infrastructures and the portal ensuring in that way an improved and updated NRGP to facilitate the user search, something that is facilitated through the fact that many organisations involved in the three developing projects are also connected to the other abovementioned projects.

### 4.3 Sustainability of the Nano Risk Governance Portal and digital infrastructure

It is recognized that the landscape for risk governance of nanomaterials is rapidly changing. Specifically, there are developments in the areas of risk governance of novel chemicals and advanced materials, where nanomaterials may be included as a separate area. This is supported by the establishment of the European partnership on the assessment risk of chemicals (PARC^20^) in 2022, which aims at tackling the challenges in the context of the new European Union Chemical Strategy for Sustainability [35], as well as the EU zero-pollution action plan [36]. As is the aim of many nano-related projects, PARC aims to build digital infrastructure(s) on a variety of topics, such as risk guidance and safe and sustainable by design [37]. In addition, various initiatives on AdMa, such as AMI2030^21^ and IM4EU [38] have identified the importance of digital infrastructures for the future of research and innovation in Europe, as part of their strategic agendas [39].

In the view of the authors, the experiences from the nano-community, including the development of this portal, provide valuable input to these overarching activities. As mentioned before, the portal is designed to be further developed, and dissemination of the content is encouraged through its open and permissive license condition. As such, it can provide a stepping stone for risk governance activities outside the nano-community.

## 5 Conclusions and future improvements

In this article, we outlined the development of a Nano Risk Governance Portal and digital infrastructure to facilitate and support the risk governance of nanomaterials. The NRGP and ordered digital infrastructure are among the primary outputs of three collaborating research projects on risk governance of nanomaterials funded through the European Union Horizon 2020 Framework Programme.

The NRGP is the result of a co-creation process of an interdisciplinary working group. Based on an initial analysis, design and development of implementation scenarios of various ambition levels, the working group decided on the most relevant scenario for the development of a portal, which intended to serve the nano-community and be relevant to varying stakeholder groups.

The portal is developed primarily as a website and is managed through an international steering committee. Its content is licensed under an open-source license, thus allowing for the possibility of further development, and maximum dissemination and uptake of its content, while removing obstacles concerning questions regarding ownership.

Within the portal, a library of tools was added, database resources were identified and linked to, in addition to information on risk governance frameworks for nanomaterials, as well as guidance on using frameworks and tools, navigating regulation and easy access to platforms and resources. As all three projects work with their own versions of a risk governance framework, the cloud platforms, framework tools, assessment tools, data development, and guidance were not included or further discussed within this article, though the portal provides direct links where possible.

The portal represents aims to be a starting point for future digital collaboration to support the nano risk governance process not only within the nano-community, but also through the ongoing and future initiatives related to AdMa. The authors therefore encourage stakeholders to link or make available their developed products, such as tools, data, models, guidances, frameworks and platforms, to the portal, by contacting the Nano-Risk Governance Portal committee using the dedicated email address^22^ available at the ‘About us’ section of the website. This will improve both its functionality and relevance, as well as increases the dissemination and uptake of individual products to the intended users of such a one-stop-shop portal.

As the creation of a digital infrastructure that satisfies multiple needs related to RG is a complicated and multi-faceted process, there are various plans for future development. Starting with the landing page of the portal, which is aimed to contain a procedure for identifying the possible users of the portal as belonging to one of several stakeholder groups – regulatory, educational, research and technology, commercial actor, or NGO. The stakeholder pre-selection will allow providing guidance for users in navigating the RG landscape and simpler selection of the most appropriate information for their research problems. This can be achieved by appropriate labelling of the content and its reproduction based on the choices and preferences of the user for its user group profile.

Further improvements of the portal could be achieved by offering fully functional and interactive data, models, tools, and guidance in the NRGP, which would be the most ambitious and most difficult task to (technically) implement into the web portal. Dynamic user interaction with tools, models and datasets, and interoperability between tools and models included on the portal would be a preferable way forward to enable the incorporation of Integrated Approaches to Testing and Assessment (IATAs) within the NRGP. This would support the FAIR and open science principles, making an increasing number of results obtained from publicly funded research available to the public. Advantages of such an approach on interoperability include that users can stay within the portal for their entire nano governance or research and development process, and even opening up for the possibility of outcome sharing between different tools included in the portal. However, there are major challenges in implementing such a solution as content cannot be easily adapted into the portal due to issues with either functionality and interoperability (e.g., tools are not available as a web service or application) or due to the license conditions of preferred material (e.g., commercial and/or protected products cannot be incorporated into the portal). Moreover, data security and confidentiality of, in particular, industries and regulatory bodies is of high concern and must be facilitated to accommodate such user needs in the future, e.g., through the incorporation of Trusted Environments [40] in the NRGP. Future initiatives could be undertaken to overcome the obstacles of such transition, in line with the expectations of the EC on research and innovation of nanomaterials and advanced materials.

## Funding

This work was supported by the Horizon 2020 Programme of the European Union under the following Grant Agreements: RiskGONE (814425), GOV4NANO (814401) and NANORIGO (814530).

## CRediT authorship contribution statement

**Panagiotis Isigonis:** Writing – review & editing, Writing – original draft, Methodology, Conceptualization. **Evert A. Bouman:** Writing – review & editing, Writing – original draft, Visualization, Methodology, Conceptualization. **Dimitra-Danai Varsou:** Writing – review & editing, Software. **Keld Alstrup Jensen:** Writing – review & editing, Methodology, Conceptualization. **Wouter Fransman:** Writing – review & editing, Methodology, Conceptualization. **Damjana Drobne:** Writing – review & editing, Methodology, Conceptualization. **Blanca Pozuelo Rollon:** Writing – review & editing, Methodology, Conceptualization. **Arantxa Ballesteros:** Writing – review & editing, Methodology, Conceptualization. **Isabel Rodríguez-Llopis:** Writing – review & editing, Methodology, Conceptualization. **Arto Säämänen:** Writing – review & editing, Methodology, Conceptualization. **Antreas Afantitis:** Writing – review & editing, Methodology, Conceptualization.

## Declaration of Competing Interest

The authors declare no conflict of interest.

## Data Availability

All data used for this research are available at www.nanoriskgov.eu

## Footnotes

1 https://www.gov4nano.eu/

2 https://nanorigo.eu/

3 https://riskgone.eu/

4 https://infrastructure.nanocommons.eu/

5 http://calibrate.risk-technologies.com/Public/index

6 https://euon.echa.europa.eu/nanodata

7 https://www.gov4nano.eu/event/nmbp-13-future-proof-approaches-for-risk-governance/

8 http://nanoriskgov.eu/regulation.html

9 http://nanoriskgov.eu/guidance.html

10 http://nanoriskgov.eu/data.html

11 http://nanoriskgov.eu/library.html

12 https://nanoinformatix-platform.greendecision.eu

13 http://cloud.nanosolveit.eu/

14 https://www.sunshine.greendecision.eu/

15 https://www.sbd4nano.eu/

16 https://www.sabyna.eu/

17 https://diamonds.tno.nl/projects/harmlesspublic

18 https://susnanofab.oppornet.com/

19 https://ec4safenano.eu-vri.eu/

20 https://www.eu-parc.eu/

21 https://www.ami2030.eu/

22 info@nanoriskgov.eu

## References

[1] EC, ‘Key Enabling Technologies (KETs) | Knowledge for policy’. Accessed: Jul. 05, 2024. [Online]. Available: 〈https://knowledge4policy.ec.europa.eu/foresight/topic/accelerating-technological-change-hyperconnectivity/key-enabling-technologies-kets_en〉.

[2] Cassee F.R. (Dec. 2024). Roadmap towards safe and sustainable advanced and innovative materials. (Outlook for 2024-2030). Comput Struct Biotechnol J.

[3] EC, . 2012. Accessed: Jul. 30, 2024. [Online]. Available: 〈https://eur-lex.europa.eu/legal-content/EN/TXT/?uri=CELEX:52012SC0288〉. COMMISSIONSTAFF WORKING PAPER Types and uses of nanomaterials, including safety aspects Accompanying the Communication from the Commission to the European Parliament, the Council and the European Economic and Social Committee on the Second Regulatory Review on Nanomaterials.

[4] Fadeel B., Pietroiusti A., Shvedova A.A. (2017). Adverse effects of engineered nanomaterials: exposure, toxicology, and impact on human health.

[5] Oberdörster G., Stone V., Donaldson K. (Jan. 2007). Toxicology of nanoparticles: a historical perspective. Nanotoxicology.

[6] Bleeker E.A.J. (Mar. 2023). Towards harmonisation of testing of nanomaterials for EU regulatory requirements on chemical safety – a proposal for further actions. Regul Toxicol Pharmacol.

[7] Grieger K. (2019). Best practices from nano-risk analysis relevant for other emerging technologies. Nat Nanotechnol.

[8] Isigonis P. (2019). Risk governance of nanomaterials: review of criteria and tools for risk communication, evaluation, and mitigation. Nanomaterials.

[9] Rasmussen K. (2023). A roadmap to strengthen standardisation efforts in risk governance of nanotechnology. NanoImpact.

[10] Groenewold M. (2024). Governance of advanced materials: shaping a safe and sustainable future. NanoImpact.

[11] Isigonis P. (2020). Risk governance of emerging technologies demonstrated in terms of its applicability to nanomaterials. Small.

[12] Lynch I. (May 2021). Jt Gloss Key Terms Relat Data Manag Knowl Infrastruct / Portal / Platf.

[13] Mullins M. (2023). Re)Conceptualizing decision-making tools in a risk governance framework for emerging technologies—the case of nanomaterials. Environ Syst Decis.

[14] A. Säämänen *et al.*, ‘Nanotechnology Risk Governance Framework (NRGF) – adaptation of the IRGC approach’, May 2021, doi: 10.5281/ZENODO.4775184.

[15] Alejandro A. (2021). Reflexive discourse analysis: a methodology for the practice of reflexivity. Eur J Int Relat.

[16] ISO, , 37000:2021, 2021. Accessed: Jul. 30, 2024. [Online]. Available: 〈https://www.iso.org/standard/65036.html〉. ISO 37000:2021 Governance of organizations — Guidance.

[17] ISO, , 21505:2017, 2017. Accessed: Jul. 30, 2024. [Online]. Available: 〈https://www.iso.org/standard/63578.html〉. ISO 21505:2017 Project, programme and portfolio management — Guidance on governance.

[18] Renn O., Roco M.C. (2006). Nanotechnology and the need for risk governance. J Nanopart Res.

[19] Drobne D. (2023). Knowledge, information, and data readiness levels (KaRLs) for risk assessment, communication, and governance of nano-, new, and other advanced materials. Glob Chall.

[20] Marcoulaki E. (2021). Blueprint for a self-sustained european centre for service provision in safe and sustainable innovation for nanotechnology. NanoImpact.

[21] Dumit V.I. (2023). From principles to reality. Fair implementation in the nanosafety community. Nano Today.

[22] Wilkinson M.D. (2016). The FAIR guiding principles for scientific data management and stewardship. Sci Data.

[23] Barker M. (2022). Introducing the FAIR principles for research software. Sci Data.

[24] OECD, . in OECD Series on the Safety of Manufactured Nanomaterials and other Advanced Materials. OECD, 2021. .Evaluation of Tools and Models for Assessing Occupational and Consumer Exposure to Manufactured Nanomaterials – Part III: Performance testing results of tools/models for consumer exposuredoi: 101787/65995f05-en.doi: 101787/65995f05-en..

[25] Gottardo S. (2021). Towards safe and sustainable innovation in nanotechnology: State-of-play for smart nanomaterials. NanoImpact.

[26] A. Porcari *et al.*, ‘State-of-the-art review on existing data on stakeholder needs in regards to support tools for safer-by-design and the overall nano-risk governance’, Deliverable D4.2, 2020. [Online]. Available: 〈https://www.gov4nano.eu/abouttheproject/project-results/〉.

[27] K.A. Jensen, B. Liguori, and C. Ribalta, ‘Implementation and operationalization of the NRGP’, Deliverable D4.7, 2023. [Online]. Available: 〈https://www.gov4nano.eu/abouttheproject/project-results/〉.

[28] Shandilya N. (2023). TRAAC framework to improve regulatory acceptance and wider usability of tools and methods for safe innovation and sustainability of manufactured nanomaterials. NanoImpact.

[29] Zins C. (2007). Conceptual approaches for defining data, information, and knowledge. J Am Soc Inf Sci.

[30] Remy F. (2022). Online]. Available. Case Stud Demonstr Nano Risk Gov Portal’ Deliv D4 6.

[31] EC, ‘Open Science - European Commission’. Accessed: Jul. 05, 2024. [Online]. Available: 〈https://research-and-innovation.ec.europa.eu/strategy/strategy-2020-2024/our-digital-future/open-science_en〉.

[32] National Academies of Sciences, Engineering, and Medicine (2018).

[33] Cheimarios N. (2021). Handbook of Functionalized Nanomaterials.

[34] Furxhi I. (2023). Status, implications and challenges of european safe and sustainable by design paradigms applicable to nanomaterials and advanced materials. RSC Sustain.

[35] EC, ‘COMMUNICATION FROM THE COMMISSION TO THE EUROPEAN PARLIAMENT, THE COUNCIL, THE EUROPEAN ECONOMIC AND SOCIAL COMMITTEE AND THE COMMITTEE OF THE REGIONS Chemicals Strategy for Sustainability Towards a Toxic-Free Environment’, 2020. Accessed: Jul. 05, 2024. [Online]. Available: 〈https://eur-lex.europa.eu/legal-content/EN/TXT/?uri=COM:2020:667:FIN〉.

[36] EC, ’. 2021. Accessed: Jul. 05, 2024. [Online]. Available: 〈https://eur-lex.europa.eu/legal-content/EN/TXT/?uri=CELEX%3A52021DC0400〉. COMMUNICATION FROM THE COMMISSION TO THE EUROPEAN PARLIAMENT, THE COUNCIL, THE EUROPEAN ECONOMIC AND SOCIAL COMMITTEE AND THE COMMITTEE OF THE REGIONS Pathway to a Healthy Planet for All EU Action Plan: ‘Towards Zero Pollution for Air, Water and Soil.

[37] Marx-Stoelting P. (2023). A walk in the PARC: developing and implementing 21st century chemical risk assessment in Europe. Arch Toxicol.

[38] EC, ‘Co-funded and co-programmed European Partnerships under the second Horizon Europe Strategic Plan.’, 2023. [Online]. Available: https://research-and-innovation.ec.europa.eu/syste m/files/2023-07/ec_rtd_candidate-list-european-partnerships.pdf.

[39] AMI2030, ‘Strategic Materials Agenda’. 2023. [Online]. Available: 〈https://www.ami2030.eu/wp-content/uploads/2023/04/Ami2030-Dossier-2.pdf〉.

[40] Soeteman-Hernandez L.G. (2019). Safe innovation approach: towards an agile system for dealing with innovations. Mater Today Commun.

